# Roles for Non-coding RNAs in Spatial Genome Organization

**DOI:** 10.3389/fcell.2019.00336

**Published:** 2019-12-19

**Authors:** Negin Khosraviani, Lauren A. Ostrowski, Karim Mekhail

**Affiliations:** ^1^Department of Laboratory Medicine and Pathobiology, MaRS Centre, Faculty of Medicine, University of Toronto, Toronto, ON, Canada; ^2^Canada Research Chairs Program, Faculty of Medicine, University of Toronto, Toronto, ON, Canada

**Keywords:** nuclear organization, non-coding RNA, DNA repeats, nucleolus, Cajal bodies (CBs)

## Abstract

Genetic loci are non-randomly arranged in the nucleus of the cell. This order, which is important to overall genome expression and stability, is maintained by a growing number of factors including the nuclear envelope, various genetic elements and dedicated protein complexes. Here, we review evidence supporting roles for non-coding RNAs (ncRNAs) in the regulation of spatial genome organization and its impact on gene expression and cell survival. Specifically, we discuss how ncRNAs from single-copy and repetitive DNA loci contribute to spatial genome organization by impacting perinuclear chromosome tethering, major nuclear compartments, chromatin looping, and various chromosomal structures. Overall, our analysis of the literature highlights central functions for ncRNAs and their transcription in the modulation of spatial genome organization with connections to human health and disease.

## Introduction

Spatial genome organization involves the 3D structure, positioning, and interactions of chromatin within the nucleus. This is a non-random process that is characterized by the regulation of various nuclear domains, topological associations, and epigenetic signatures. For example, decondensed euchromatin domains, which include active enhancer elements and are generally conducive to transcription, are found preferentially within the nuclear interior. On the other hand, heterochromatin domains are densely packed chromosome regions that are occupied by gene-silencing histone marks, which include histone H3 methylated on Lysine 27 (H3K27me3) or Lysine 9 (H3K9me2 and H3K9me3) (Rice and Allis, 2001; Richards and Elgin, 2002). Such heterochromatin domains are preferentially located near the nuclear periphery or a major nuclear compartment called the nucleolus.

In fact, the nuclear genome is generally arranged within several cytologically distinct compartments. In addition to the prominent nucleolus, other nuclear compartments include the Cajal bodies, speckles, paraspeckles, and histone locus bodies. Nuclear compartments generally form via dynamic self-organization of their different constituents at sites of gene clusters (Mao et al., 2011; Sleeman and Trinkle-Mulcahy, 2014; Wang et al., 2016). For example, nucleoli encompass the tandem ribosomal DNA (rDNA) repeats while histone locus bodies form around the histone-encoding gene clusters. Early studies identified a role for molecular crowding in the formation of some nuclear compartments (Richter et al., 2007; Cho and Kim, 2012). High concentrations of macromolecules in a local environment creates crowding and promotes formation of weak non-covalent bonds between the macromolecules, thereby forming membrane-less nuclear compartments. Consistent with this notion, the formation of several nuclear compartments is driven by liquid-liquid phase separation (Zhu and Brangwynne, 2015; Hall et al., 2019). Importantly, the three-dimensional organization of chromatin into these nuclear compartments often underlies the expression and stability of the various genetic loci that are harbored within such nuclear bodies. For example, actively transcribed genes often associate with the periphery of nuclear speckles, which are sites of RNA processing (Hu et al., 2019). Disruption of nuclear speckles changes gene expression profiles by decreasing intrachromosomal interactions between active chromatin regions.

In addition to the formation of cytologically distinct nuclear compartments, the genome is organized into topologically associated domains (TADs) (Dixon et al., 2012), which can be viewed as three-dimensional building blocks of looped chromatin domains (Lieberman-Aiden et al., 2009; Dixon et al., 2012). TADs are present in the genomes of several eukaryotes including *Drosophila* (Sexton et al., 2012), mice (Krefting et al., 2018) and humans (Lieberman-Aiden et al., 2009), and are categorized into type A (active genes) and type B (inactive genes) compartments. TADs can regulate transcription by acting as insulators, preventing the spread of euchromatin or heterochromatin marks and regulating enhancer-promoter interactions.

Topologically associated domains are built or defined by their associated proteins, which include the cohesin complex, condensin complex and CCCTCF binding factor (CTCF) which binds DNA in a sequence-specific manner (Dixon et al., 2012; Zuin et al., 2014). Cohesin and condensin are ring-shaped protein complexes that bind chromatin independently of the DNA sequence and mediate chromatin looping, bringing distant DNA sequences along the linear genome into close proximity within the 3D space of the nucleus (Nuebler et al., 2018). The cohesin and condensin complexes, which are composed of structural maintenance of chromosome (SMC) proteins, extrude the DNA into loops through an ATP hydrolysis-dependent mechanism (Burmann et al., 2017; Diebold-Durand et al., 2017; Ganji et al., 2018). Cohesin loading onto chromatin is mediated by the loading factor NIPBL, the absence of which results in the loss of local TAD patterns (Schwarzer et al., 2017). The DNA is extruded until cohesin reaches a boundary element or insulator such as CTCF (Nuebler et al., 2018; Vian et al., 2018). CTCF is a DNA binding protein that mostly associates with TAD boundary regions, insulator sequences, and imprinting control regions (Rao et al., 2014; Sanborn et al., 2015). CTCF is responsible for the majority of chromatin loops across the human genome and is thus an important regulator of spatial genome organization.

Another regulator of spatial genome organization is the nuclear envelope, which harbors the inner nuclear membrane (INM) proteins and nuclear pore complexes (NPCs) and is lined by the nuclear lamina (NL), which is a meshwork of lamin and lamin-associated proteins. The nuclear lamins are important regulators of chromatin organization (Kind et al., 2015). Genes that are activated for transcription are commonly repositioned from the NL to either the nuclear interior or closer to NPCs. Regions of the chromatin that interact with the lamina are referred to as lamina associated domains (LADs), and this association is mediated by lamin-associated proteins. In mammals (Guelen et al., 2008), nematodes (Ikegami et al., 2010) and flies (Pickersgill et al., 2006; van Bemmel et al., 2010), LADs mostly harbor silent or weakly expressed genes, and contain heterochromatin marks such as H3K9me3 and H3K9me2 (Casolari et al., 2004; Wen et al., 2009), whereas budding yeast has no lamina or LADs and its genome is instead organized into gene crumples and directly tethered to INM or NPC proteins (Taddei et al., 2006; Mekhail et al., 2008; Hsieh et al., 2015). In *Drosophila* cells, NL disruption alters LAD composition such that there is more histone H3 acetylated on Lysine 9 (H3K9Ac) and less chromatin compaction (Ulianov et al., 2019). Furthermore, association of chromosomes with the nuclear lamina limits their mobility within the nucleus (Wang H. et al., 2018). In addition, studies in different organisms revealed that NPCs can regulate chromatin structure and function (Dilworth et al., 2005; Brown et al., 2008; Mekhail and Moazed, 2010). For example, the nucleoporins from which NPCs are built can associate with the promoters of active genes in yeast, thereby regulating gene expression (Schmid et al., 2006).

In addition to nuclear compartments, TADs/LADs, the nuclear envelope and their associated protein complexes, non-coding RNAs (ncRNAs) have emerged as major regulators of spatial genome organization. ncRNAs are RNA molecules that are not translated into proteins. ncRNAs are categorized based on their size – long (>200 bp) and short (<200 bp) – and are implicated in numerous cellular processes including transcription, mRNA splicing, and protein translation (Mortazavi et al., 2008; Khalil et al., 2009; Palazzo and Lee, 2015). ncRNAs emerging from within a given genetic locus can regulate transcription at the same locus (*cis*) or elsewhere in the genome (*trans*). Here we review ncRNAs that emerge from single-copy DNA loci or repetitive DNA loci and have diverse roles in spatial genome organization, thus impacting gene expression and stability. Collectively, ncRNAs impact spatial genome organization by modulating perinuclear chromosome tethering, the formation of major nuclear compartments, chromatin looping and various chromosomal structures. These roles of ncRNAs often intersect with various other regulators of genome structure and function.

## Non-Coding RNA at Single Copy Loci

**FIGURE 1:**
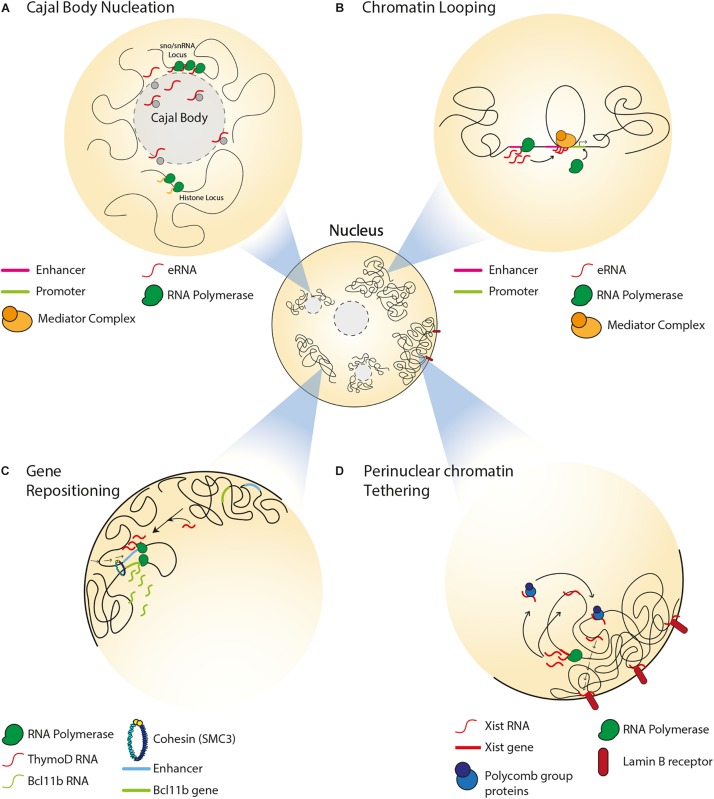
Spatial organization of single copy loci by ncRNAs. **(A)** Transcription of snRNA genes and interaction between intron-encoded snoRNA/snRNAs with coilin mediate Cajal body formation. Cajal bodies associate with sn/snoRNA and histone gene loci and regulate their gene expression. **(B)** Transcription of an enhancer can produce eRNAs, which associate with the mediator complex and enable chromatin looping, thereby driving enhancer-promoter interaction. **(C)** In the absence of the *ThymoD* ncRNA, the enhancer for the *BCL11B* locus is at nuclear periphery. *ThymoD* ncRNA mediates enhancer repositioning away from the nuclear periphery and drives chromatin looping of the enhancer bringing it in close proximity to the *BCL11B* locus, thereby allowing for the transcriptional activation of this locus. **(D)** In the somatic tissues of placental mammals, *Xist* lncRNA tethers the inactive X chromosome to the nuclear lamina by interacting with lamin B receptor. *Xist* interacts with polycomb proteins to establish the heterochromatin state of the inactive X chromosome. *Xist* also mediates relocation of active genes from the surface of the X chromosome to its interior.

Single copy loci include genes required for cell function and survival and can give rise to ncRNAs that regulate higher order chromatin structure and positioning (Figure 1). ncRNAs and their active transcription can mediate chromatin looping to bring distant DNA regions into close proximity and reposition genetic loci to regulate their expression. Nuclear bodies, such as Cajal bodies and paraspeckles, are formed by ncRNA transcription and can regulate the localization or sequestration of transcriptional regulators. Furthermore, ncRNAs play roles in organismal development by regulating the subnuclear positioning and transcriptional status of the X chromosome, HOX genes and *Kcnq1* genes. In this section we discuss roles for ncRNAs and their transcription in the control of spatial gene positioning, chromatin remodeling and nuclear compartmentalization.

### ncRNAs in the Formation and Maintenance of Nuclear Compartments

Non-coding RNAs can impact the structure and function of nuclear compartments such as Cajal bodies. The latter are involved in various processes including telomerase biogenesis, 3′-end processing of histone pre-mRNAs, as well as the processing, assembly and maturation of spliceosomal small nuclear ribonucleoproteins (snRNPs) (Sawyer et al., 2016). Cajal bodies associate with small nuclear and nucleolar RNA (sn/snoRNA) gene loci, such that these genes form intra- and inter-chromosomal clusters around the bodies (Figure 1A) (Wang et al., 2016). In fact, the formation of Cajal bodies is itself mediated by the transcription of snRNA genes (Kaiser et al., 2008) and by interactions between intron-encoded snoRNA/snRNAs and a protein called coilin (Kaiser et al., 2008; Machyna et al., 2014). This is in accordance with studies reporting the loss of Cajal bodies during mitosis and their reformation during early G1 upon the resumption of transcription (Carmo-Fonseca et al., 1993; Strzelecka et al., 2010). Furthermore, these ncRNA-dependent Cajal bodies are responsible for the spatial organization and expression of other types of genes, including those encoding for histones or pre-mRNA splicing factors (Sawyer et al., 2016; Wang et al., 2016; Wang H. et al., 2018).

Paraspeckles are nuclear bodies that form in response to environmental stress at and around the *NEAT1* gene (nuclear enriched abundant transcript 1), which is transcribed into the long ncRNAs (lncRNAs) *NEAT1_1* (Men ε) and *NEAT1_2* (Men β) (Sunwoo et al., 2009). These lncRNAs and their ongoing transcription are required for the nucleation and maintenance of these nuclear compartments (Shevtsov and Dundr, 2011). Transcriptional upregulation of *NEAT1* increases paraspeckle size and sequestration of paraspeckle-associated transcriptional regulators, such as the splicing factor proline/glutamine-rich (SFPQ) (Hirose et al., 2014). In contrast, repression of *NEAT1* disrupts paraspeckles, releases paraspeckle-associated proteins into the nucleoplasm and hyper-induces the transcription of various genes including *ADARB2* (adenosine deaminase RNA-specific B2), which is involved in RNA editing (Clemson et al., 2009; Mao et al., 2011; Hirose et al., 2014; Imamura et al., 2014).

*NEAT1* can also regulate the subnuclear localization of growth control genes by associating with Polycomb 2 protein (Pc2), a key subunit of the chromatin-repressive PRC1 complex (Yang et al., 2011). Methylation/demethylation cycles of Pc2 dictate its association with two ncRNAs, *TUG1* (Taurine up-regulated 1) and *NEAT1*, which are found in two distinct nuclear bodies. Methylated Pc2 preferentially interacts with the *TUG1* ncRNA within the transcriptionally repressive Polycomb nuclear bodies, thereby silencing the Pc2-associated growth control genes. On the other hand, demethylation of Pc2 results in its preferential interaction with *NEAT1*, which relocates Pc2 together with its associated growth control genes to inter-chromosomal granules within which the genes can be actively transcribed.

*NEAT1* is commonly induced upon viral infection and can regulate the transcriptional activation of various antiviral genes (Ma et al., 2017). The splicing factor SFPQ is a transcriptional repressor of the antiviral gene *IL-8.* Recently, *NEAT1* has been shown to mediate the relocation of SFPQ from the *IL-8* promoter to paraspeckles, thereby activating *IL-8* gene expression (Imamura et al., 2014). Paraspeckles and *NEAT1* have also been linked to cancer biology, where they can have both oncogenic and tumor suppressive roles. In some cancers, the upregulation of *NEAT1* and associated paraspeckles can be driven by tumor microenvironmental conditions and estrogen receptor stimulation, respectively (Chakravarty et al., 2014; Choudhry et al., 2015). This upregulation is associated with increases in active epigenetic marks and cellular proliferation. Surprisingly, in some types of cancer, upregulation of *NEAT1* and paraspeckles prevented cellular transformation and tumorigenesis (Adriaens et al., 2016). Overall, these findings highlight functional connections between ncRNAs and nuclear compartments. These studies also underscore the importance of understanding the exact roles that ncRNAs can exert within different biological and clinical settings.

### ncRNAs and Chromatin Looping

Non-coding RNAs can regulate gene expression by mediating chromatin remodeling between enhancers and promoters. Transcription of enhancers in mammalian cells can give rise to a type of ncRNA that is referred to as enhancer RNA (eRNA), which can bring an enhancer and promoter in close proximity by mediating the formation of a DNA loop, and associate with mediator complexes to drive the expression of target genes (Figure 1B) (Kim et al., 2010; Orom et al., 2010). For example, activation of estrogen receptor-α induces the transcription of eRNAs that mediate chromatin looping, thereby driving transcription-inducing enhancer-promoter interactions at target genes (Li W. et al., 2013). Another class of ncRNAs, which is known as ncRNA-activating (ncRNA-a), has a function similar to that of eRNA (Lai et al., 2013; Li W. et al., 2013). These ncRNA-a species activate their neighboring genes by associating with the mediator complex and enabling chromatin looping in *cis*. This 3D chromatin configuration and gene expression are reduced upon disruption of ncRNA-a species or mediator, suggesting the dependence of chromatin loop structure and function on interactions between ncRNA-a and mediator.

The active transcription of ncRNAs can also result in the looping of DNA, bringing gene loci in close proximity or blocking transcription of distant genes. In the plant *Arabidopsis thaliana*, transcription of the ncRNA *APOLO* forms a chromatin loop encompassing the promoter of its neighboring gene, *PID* (Ariel et al., 2014), which is the key regulator of polar auxin transport and root development (Benjamins et al., 2001). This *APOLO*-mediated 3D chromatin configuration, which is also influenced by PRC1 and PRC2 (polycomb repressive complex 1 and 2), limits the access of Pol II to the *PID* promoter, thereby regulating the transcriptional activity of this gene (Ariel et al., 2014). Disruption of the *APOLO*-dependent expression of *PID* results in defects in root development, highlighting the importance of ncRNA-mediated chromatin remodeling to plant growth and development (Benjamins et al., 2001).

Intergenic transcription-driven chromatin looping is also implicated in lymphocyte development. In developing B cells, V(D)J recombination is required for the assembly of antigen receptors (Alt et al., 1984; Sayegh et al., 2005). Importantly, V(D)J recombination requires changes to the 3D configuration of the immunoglobulin heavy locus (*Igh*) in order to bring the V_H_, D_H_ and J_H_ genes in close proximity, which in turn allows the genetic rearrangements to occur (Kosak et al., 2002; Medvedovic et al., 2013). Prior to rearrangement, non-coding transcription at this locus occurs at the V_H_ intergenic region in the antisense orientation (Yancopoulos and Alt, 1985). This intergenic region contains Pax5-activated intergenic repeat (PAIR) elements (Fuxa et al., 2004), which are transcriptionally upregulated in the absence of CTCF (Degner et al., 2009). Antisense transcription of these PAIR elements in pro-B cells mediates long-distance interaction with the E_μ_ region on the *Igh* locus (Verma-Gaur et al., 2012). The resulting DNA looping brings the distal V_H_ into close proximity with DJ_H_ and allows for V_H_ to DJ_H_ recombination. These DNA loops are not observed in the absence of ncRNA transcription, highlighting the importance of active ncRNA transcription to V(D)J recombination and its role in B cell development.

In developing T cells, expression of BCL11B (BAF Chromatin Remodeling Complex Subunit BCL11B) promotes expression of T-lineage-specific genes and suppresses expression of the genes associated with alternative cell fates (Li et al., 2010). Activation of BCL11B expression is mediated by an enhancer that is located at the so-called intergenic control region (ICR) (Li L. et al., 2013). Repositioning of the enhancer from the nuclear lamina to the interior allows for the transcriptional activation of *BCL11B* (Figure 1C) (Isoda et al., 2017). Importantly, this relocation within nuclear space is mediated by transcription of the ncRNA *ThymoD* (thymocyte differentiation factor), which mediates DNA demethylation at CTCF binding sites and subsequent activation of CTCF/cohesin-dependent chromatin looping.

### ncRNAs and X Chromosome Silencing and Positioning

One of the well-studied ncRNAs implicated in mammalian 3D genome organization is *Xist* (X inactive specific transcript), a 17 kb lncRNA that mediates inactivation of one of the X chromosomes during early female embryonic development (Brown et al., 1992). *Xist* is specifically transcribed from the inactive X chromosome. *Xist* occupies inactive regions of the X chromosome before spreading across transcriptionally active regions and initiating their inactivation. Subsequently, the inactive X chromosome forms a heterochromatic structure, which is referred to as Barr body and is found at the perinuclear and perinucleolar regions, where transcription silencing machineries are enriched (Zhang et al., 2007). Of note, tethering of the inactive X chromosome to the nuclear lamina is the result of interactions between *Xist* and the INM-embedded lamin B receptor (Figure 1D) (Chen et al., 2016). This interaction repositions transcriptionally active DNA regions of the X chromosome in close proximity with *Xist* and its transcriptional silencing domain, thereby promoting the spread of *Xist* across the chromosome. In female embryonic stem cells, the spreading of *Xist* along an X chromosome results in the establishment of polycomb group proteins-dependent heterochromatin and exclusion of transcription machineries (Figure 1D) (Plath et al., 2003; Okamoto et al., 2004; Chaumeil et al., 2006; Schoeftner et al., 2006). During this process, active genes that were once on the surface of the X chromosome relocate to the interior, forming *Xist*-containing transcriptionally silent domains (Chaumeil et al., 2006). Furthermore, *Xist* maintains this heterochromatic nuclear compartment by acting in *cis* to repel cohesin and other chromatin looping factors that typically facilitate gene expression (Minajigi et al., 2015). Consequently, compared to the active X chromosome, the inactive X chromosome is devoid of TADs, which can nonetheless be re-established upon depletion of *Xist* and restoration of cohesin loading (Nora et al., 2012). Taken together, these findings highlight how the *Xist* lncRNA mediates mammalian X chromosome inactivation through the formation of perinuclear heterochromatin domains and the exclusion of factors that can promote chromatin looping and gene expression.

The expression of *Xist* on the active X chromosome is regulated by another lncRNA, *Tsix*, which is transcribed antisense to *Xist* (Stavropoulos et al., 2001). Transcription of *Tsix* represses *Xist* expression in *cis* through epigenetic processes (Stavropoulos et al., 2001; Shibata and Lee, 2004). In mouse embryonic stem cells, the X chromosome lacking *Tsix* transcription was non-randomly inactivated (Lee and Lu, 1999; Luikenhuis et al., 2001), and induction of *Tsix* transcription resulted in targeted X chromosome activation (Luikenhuis et al., 2001). Therefore, *Tsix* and *Xist* play antagonistic roles in regulating X chromosome inactivation during embryonic stem cell differentiation.

In addition to *Xist* and *Tsix*, *Firre* (functional intergenic repeating element) is another lncRNA that is transcribed from a locus on the X chromosome (Hacisuleyman et al., 2014). *Firre* can maintain the silencing of the X chromosome by tethering it to the perinucleolar compartment (Yang et al., 2015). In addition, *Firre* interacts with the nuclear matrix factor hnRNPU and colocalizes with five distinct *trans*-chromosomal loci, which reside in spatial proximity to the *Firre* locus. This colocalization is lost in the absence of *Firre*, suggesting a role of this ncRNA in the establishment of higher order chromosomal architectures within nuclear space.

Typically, cells randomly choose whether the maternal or paternal X chromosome is inactivated. However, under certain circumstances, there can be bias toward one parental X chromosome. Such a bias is referred to as skewed X inactivation. In females, this can result in diseases such as Duchenne muscular dystrophy and hemophilia A (Yoshioka et al., 1998; Renault et al., 2007). Incomplete silencing of the X chromosome can also result in skewed X inactivation since some genes manage to evade silencing and remain therefore expressed. For example, escape of the steroid sulfatase locus from silencing can trigger X-linked ichthyosis, which is a group of diseases characterized by very dry skin (Hernandez-Martin et al., 1999). Thus, ncRNAs operate at the interface of spatial genome organization and epigenetic silencing to mediate X chromosome inactivation, the dysregulation of which underlies different human diseases.

### ncRNAs and the Spatial Organization of Developmental Genes

During vertebrate development, ncRNAs can regulate the spatial organization of gene clusters, such as the *HOX* genes (Flyamer et al., 2017). *HOX* genes, which are homeotic genes involved in antero-posterior body axis development in vertebrates, are found on four spatially clustered chromosomal loci (*HOXA*, *HOXB*, *HOXC*, and *HOXD*). The genes are separated into distinct topological compartments based on their transcriptional profile, and during development, there exists a dynamic switch between these topological domains (Noordermeer et al., 2011). This higher order structure of the *HOX* gene clusters is regulated by intergenic ncRNAs (Wang et al., 2011). For example, the ncRNA *HOTTIP* (*HOXA* transcript at the distal tip) is transcribed from the 5′ edge of the *HOXA* locus and is required for maintaining the compartmentalization of the locus. *HOTTIP* can associate with and target the WD repeat mixed lineage leukemia (WDR-MLL) complex across the *HOXA* locus to yield active histone marks. This in return maintains the active state of some of the *HOXA* genes. *HOTTIP* also physically associates with CTCF, which can bind to six conserved binding sites at *HOXA* and serve as an insulator (Wang F. et al., 2018). This contributes to the discrete expression profile of genes across the *HOXA* locus. The dynamic 3D architecture of these gene clusters is important as it dictates the transcriptional profile of the *HOX* genes during development. Dysregulation of *HOX* gene expression can abrogate limb and skeletal development in murine and *Drosophila* embryos (Di-Poi et al., 2010; Andrey et al., 2013). Therefore, regulation of the spatial organization of HOX genes by ncRNAs is important for organismal development.

Another critical component of development is known as genetic imprinting, which consists of the silencing of one parental allele. Imprinted genes tend to spatially cluster and this allows for their coordinated regulation during development. lncRNAs have been shown to regulate the expression and large-scale chromatin structure of these genes through interaction with histone modifying proteins and chromatin looping (Umlauf et al., 2004; Terranova et al., 2008; Zhang et al., 2014). In early mammalian embryos, the *Kcnq1* genes cluster into a compact subnuclear compartment, devoid of transcriptional activity (Verona et al., 2003; Lewis et al., 2006). This nuclear compartment is enriched with repressive histone marks and silencing protein complexes such as polycomb proteins (Umlauf et al., 2004; Terranova et al., 2008). Formation of this higher order repressive domain and its localization within the perinucleolar compartment is mediated by the *Kcnq1ot1* ncRNA, which associates with the H3K9me3 repressive histone mark and polycomb proteins (Mohammad et al., 2008; Pandey et al., 2008; Terranova et al., 2008). *Kcnq1ot1* is an antisense ncRNA (∼100 kb) that is transcribed from the intronic region of the *Kcnq1* locus of one of the parental chromosomes. Deletion of *Kcnq1ot1* results in expression of the parental allele that is normally silent (Mancini-Dinardo et al., 2006). More recently, this ncRNA has been shown to directly interact with the chromosome, through its 5′ terminal region, in order to mediate intrachromosomal looping between the *Kcnq1* promoter and *Kcnq1ot1* promoter KvDMR (Zhang et al., 2014). These promoters are 200 kb apart in the linear genome (Zhang et al., 2014). However, promoter looping results in the imprinting of the *Kcnq1* cluster, or its allelic silencing. Deletion of KvDMR can result in biallelic expression of maternal-specific genes in the *Kcnq1* cluster and growth deficiency in mice (Fitzpatrick et al., 2002; Shin et al., 2008). In humans, loss of imprinting can lead to Beckwith–Wiedemann syndrome, which is associated with cancer growth and progression (Lee et al., 1999; Fitzpatrick et al., 2002; Valente et al., 2019). Therefore, regulation of the spatial organization of the *Kcnq1* gene cluster by *Kcnq1ot1* is important for mammalian genetic imprinting and healthy development. Overall, these findings suggest that ncRNAs play a role in regulating gene expression during development via establishment of nuclear compartments and regulation of locus positioning within nuclear space.

Taken together, ncRNAs from single-copy loci contribute to spatial genome organization through chromatin remodeling, nuclear compartmentalization and the subnuclear positioning of various genes within nuclear space. These roles of ncRNA help mediate cellular processes that are central to the proper control of gene expression, genome stability, development, and health.

## Non-Coding RNA at Repetitive DNA Loci

Eukaryotic genomes are largely composed of repetitive DNA sequences that can be generally classified as tandem or interspersed repeats. Tandem repeats include satellite and minisatellite repeats (e.g., centromeres) as well as microsatellite repeats (telomeres). Interspersed repeats include transposable elements that are either retrotransposons or DNA transposons. Retrotransposons include LTR-retrotransposons such as HERV and non-LTR retrotransposons such as SINEs (e.g., Alu), LINEs (e.g., LINE-1) or SVAs. It is also important to note that some types of repeats such as human ribosomal DNA (rDNA) can be arranged in tandem repeats that are interspersed throughout the linear genome. Regardless of their relative genomic location, DNA repeats are often clustered within nuclear space. This can facilitate their transcriptional co-regulation, minimize their potential deleterious interaction with the rest of the genome and control their exposure to potentially genome-destabilizing DNA recombination and repair machineries.

Repetitive DNA sequences are non-randomly arranged within the nucleus. For example, rDNA repeats are physically sequestered in the nucleolar compartment of the nucleus. This sequestration can be driven by inter- or intra-chromosomal interactions, or even direct tethering to the nuclear envelope in some organisms (O’Sullivan et al., 2009; Mekhail and Moazed, 2010; Chan et al., 2011; Hult et al., 2017). Telomeres, which are at the ends of linear chromosomes, often colocalize within PML bodies (Chang et al., 2013) at the nuclear interior or within telomeric clusters or bouquets at the nuclear periphery (Gotta et al., 1996). In budding yeast, the Transposons of Yeast 1 (Ty1) retrotransposons cluster within or near nucleoli (O’Sullivan et al., 2009), while centromeres cluster at the yeast spindle pole body (Jin et al., 2000). Importantly, several ncRNAs from repetitive DNA loci have emerged as major players that mediate crosstalk between spatial genome organization, expression and stability. Here were review such ncRNAs, which emerge from rDNA repeats, telomeric regions, transposable elements, and centromeres.

### Non-coding RNAs in rDNA Structure and Function

**FIGURE 2:**
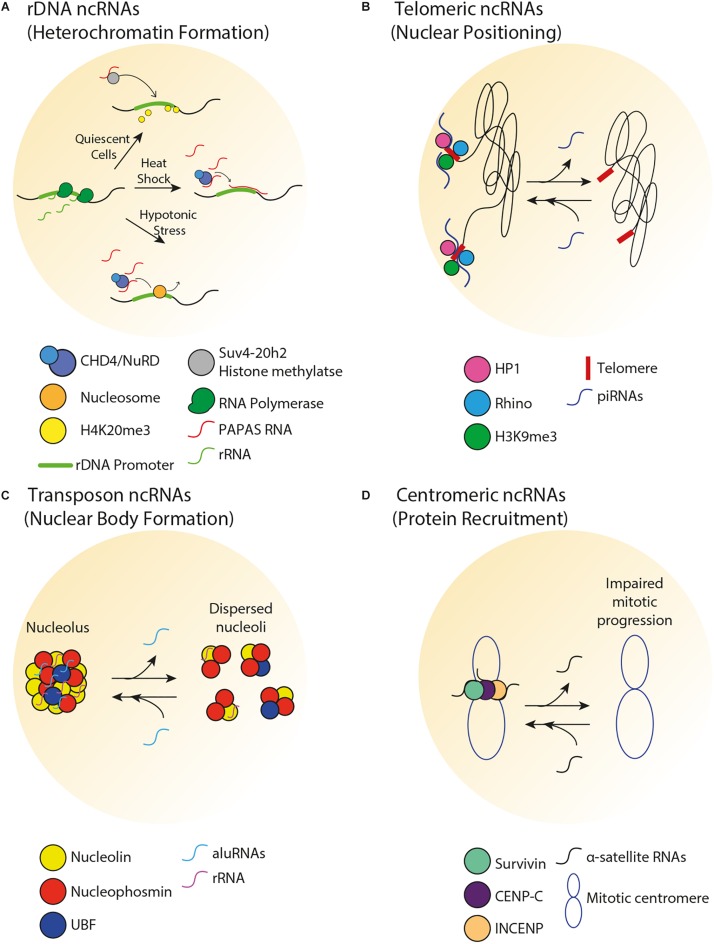
Spatial organization of repetitive DNA loci by ncRNA. **(A)** In human cells, there is an inverse correlation between PAPAS and pre-rRNA levels. In quiescent cells, PAPAS binds to the Suv4-20h2 histone methyltransferase and directs it to the rDNA promoter for H4K20me3-dependent repression. Upon heat shock, PAPAS hybridizes with the rDNA promoter and recruits the CHD4/NuRD complex, thereby preventing rDNA transcription. Upon hypotonic stress, upregulation of PAPAS recruits the CHD4/NuRD complex to reposition rDNA promoter-bound nucleosome to the off position, thereby halting pre-rRNA synthesis. **(B)** In germline tissues of flies, piRNAs transcribed from the telomeric region mediate perinuclear positioning of telomeres and promote HP1, Rhino, and H3K9me3 enrichments at telomeres. **(C)** In human cells, aluRNAs enriched in the nucleolus interact with nucleolin to maintain nucleolar structure and function. **(D)** In human cells, α-satellite RNAs associate with and promote the centromeric enrichment of Survivin, CENP-C, and INCENP in order to maintain centromere stability.

Non-coding RNAs can play a role in the spatial organization and function of rDNA through the modulation of heterochromatin formation. Transcription of rDNA into ribosomal RNA (rRNA) molecules is dependent on the varying demand for protein synthesis that cells experience in response to intracellular signals or environmental stimuli. Therefore, despite the existence of 100s of rDNA repeats in eukaryotes, only a fraction of rDNA units are transcribed, while the remainder of the repeats is epigenetically silenced. Transcriptionally active rDNA units are marked by DNA hypomethylation, H4Ac and H3K4me2, whereas inactive rDNA units are marked by promoter hypermethylation, histone H4 hypoacetylation and methylation of H3K9, H3K27, and H4K20 (Santoro et al., 2002). Deposition of these marks is facilitated by the nucleolar remodeling complex (NoRC), which guides chromatin remodeling proteins to the rDNA and other loci. In mice, NoRC is recruited to nucleoli through interaction between the large subunit of NoRC (TIP5) and promoter-associated RNAs (pRNAs) that overlap the rDNA promoter (Mayer et al., 2006). A class of pRNAs termed PAPAS (promoter and pre-rRNA antisense) covers the rDNA promoter, and levels of PAPAS generally reflect the physiological state of the cell, such that there is an anti-correlation between PAPAS and pre-rRNA levels (Figure 2A) (Bierhoff et al., 2010). In quiescent mammalian cells, PAPAS is induced, binds to the histone methyltransferase Suv4-20h2, targets it to the rDNA promoter and downregulates rRNA transcription through enhanced H4K20me3 (Bierhoff et al., 2014). In addition, upon heat shock, upregulation of PAPAS attenuates pre-rRNA synthesis by recruiting another chromatin remodeling complex named CHD4/NuRD to the mammalian rDNA promoter (Zhao et al., 2018). On another front, in mammalian cells under hypotonic stress conditions, PAPAS upregulation recruits NuRD to reposition the rDNA promoter-bound nucleosome to the “off” position, thereby halting pre-rRNA synthesis (Zhao et al., 2016). Interestingly, pRNA-dependent heterochromatin formation at rDNA has also been shown to initiate the downstream establishment of heterochromatic structures at genomic regions that are in close proximity but lie outside of the murine nucleolus (Savic et al., 2014). Taken together, these studies reveal that under different environmental conditions, promoter-associated ncRNAs from repetitive loci can silence gene expression in *cis* through various processes. Future studies should explore how such ncRNAs are induced under different environmental conditions.

The nucleolus typically exhibits a phase separation-driven tripartite organization into a fibrillar centre (FC), dense fibrillar component (DFC), and granular component (GC) (Feric et al., 2016; Hall et al., 2019). Upon exposure to environmental stresses including heat shock or acidosis, a couple of ncRNAs induced from the mammalian rDNA intergenic spacer (IGS) dissolve this tripartite organization, structurally remodeling the nucleolus into a so-called “protein detention centre” (DC) (Mekhail et al., 2005; Jacob et al., 2013). The DC is suggested to be spatially, dynamically and biochemically distinct from the standard tripartite domains (Jacob et al., 2013). This structural remodeling of the mammalian nucleolus can arrest rRNA synthesis and create a hub for immobilized proteins, effectively mediating their nucleolar sequestration and functional inactivation (Audas et al., 2012). Upon removal of the environmental stressor, the ncRNAs are repressed, DC is dissolved and tripartite nucleolar organization is re-established (Jacob et al., 2013). Thus, ncRNAs spatially remodel the nucleolus during stress. Importantly, future studies should explore how cells control the generation and function of such intergenic ncRNAs under varying environmental conditions.

The organization of rDNA repeat regions into epigenetically silent chromatin structures is essential to proper cellular function and alterations in this organization may be associated with human disease. For example, rDNA hypermethylation is characteristic of early Alzheimer’s disease (Pietrzak et al., 2011), upregulation of rRNA expression is characteristic of tumor cells (White, 2005; Montanaro et al., 2008; Bywater et al., 2013) and rRNA dysfunction is linked to a group of genetic diseases known as ribosomopathies (Narla and Ebert, 2010; Nakhoul et al., 2014). In addition, in yeast, the dysregulation of IGS ncRNAs at rDNA repeats has been associated with premature aging through three distinct mechanisms. First, loss of IGS silencing leads to the upregulation of IGS ncRNAs, which displace cohesin complexes, triggering rDNA instability and premature aging (Saka et al., 2013). Second, IGS ncRNAs are prone to the formation of DNA replication-blocking RNA–DNA hybrid-containing structures called R-loops (Salvi et al., 2014). When these structures accumulate, as in some yeast mutants, unequal sister chromatid exchange events occur within the rDNA repeats, leading to chromosome instability and premature cellular aging (Salvi et al., 2014). Lastly, in yeast genetic models of neurodegenerative diseases, hyper-reductions in IGS ncRNA levels can lead to rDNA copy number instability and premature cellular aging (Ostrowski et al., 2018). Thus, ncRNAs that play important roles in the epigenetic silencing and organization of rDNA repeats can impact processes underlying organismal health span.

### Crosstalk Between Telomeric ncRNAs, Heterochromatin, and Subnuclear Positioning

The telomeres at the end of linear chromosomes are often heterochromatic. In vertebrates, telomeres are composed of hexameric 5′-TTAGGG-3′ repeats that are flanked by repetitive subtelomeric regions. Telomeric and subtelomeric repeats exhibit heterochromatic marks (H3K9me3, H4K20me3, and hypoacetylation of H3 and H4). Loss of heterochromatin disrupts telomere length control, increases telomeric recombination and promotes premature cellular senescence (Garcia-Cao et al., 2004; Benetti et al., 2007). Interestingly, the establishment of telomeric heterochromatin is influenced by a type of ncRNA called telomeric repeat-containing RNA (TERRA), which is composed of UUAGGG repeats (Nergadze et al., 2009). TERRA transcription typically initiates from subtelomeric CpG islands and proceeds to chromosomal ends. Several lines of evidence support a role for TERRA in the regulation of heterochromatin and other structures near chromosome ends. First, TERRA associates with TIP5 and subsequently recruits NoRC and the histone-modifying enzymes Suv4-20h2 and SIRT6 to human telomeres (Postepska-Igielska et al., 2013). Second, loss of human TERRA decreases telomeric H3K9me3 and HP1 enrichments and induces the DNA damage response (Blasco, 2007; Deng et al., 2009). Third, TERRA facilitates heterochromatin-promoting interactions between the human Shelterin complex, HP1 and the origin recognition complex (Deng et al., 2009). Fourth, TERRA transcription initiates at subtelomeric CTCF-binding sites, tentatively suggesting that the transcription of TERRA is itself spatially regulated by chromosome looping (Beishline et al., 2017). ncRNAs also regulate telomeric heterochromatin formation in non-vertebrate species. For example, small ncRNAs are implicated in heterochromatin formation at fission yeast telomeres (Cao et al., 2009). Together, these studies highlight a role for telomeric ncRNAs in the promotion of local heterochromatin structures and consequent prevention of premature cellular senescence. Importantly, there is crosstalk between telomeric heterochromatin and the subnuclear positioning of telomeres. For example, in budding yeast, the constitutive co-localization of telomeres in a handful of clusters at the nuclear periphery increases the local concentration of chromatin silencing factors, reinforcing telomeric heterochromatin and limiting access to the potentially genome-destabilizing recombination machinery (Therizols et al., 2006; Schober et al., 2009; Chan et al., 2011). In the fly germline, loss of some PIWI-interacting RNAs (piRNAs) that are typically transcribed from telomeric regions decreased perinuclear telomere positioning and lowered the local enrichment of HP1, Rhino, and H3K9me3 (Figure 2B) (Radion et al., 2018).

Connections exist between telomere malfunction and disease. The aberrant loss of telomeric heterochromatin can trigger telomeric DNA damage responses and recombination events, which are associated with several diseases (Hagelstrom et al., 2010). The accumulation of TERRA-associated R-loops drives telomere instability in the rare autosomal recessive syndrome ICF (immunodeficiency, centromeric instability, and facial anomalies; Sagie et al., 2017). Similarly, in budding yeast, elevated TERRA levels can promote premature senescence (Wanat et al., 2018). On another front, various changes to TERRA levels are linked to cancer (Artandi and DePinho, 2010), dyskeratosis congenita (Armanios et al., 2009; Gu et al., 2009; Mason and Bessler, 2011) and aplastic anemia (Armanios et al., 2009; O’Sullivan and Karlseder, 2010; Armanios, 2012). We refer the reader elsewhere for a full review on the emerging connections between telomeric ncRNAs and disease (Maicher et al., 2012). Taken together, these studies suggest that telomeric ncRNAs modulate heterochromatin formation and subnuclear positioning at telomeres to promote health and longevity.

### Transposable Elements

Similar to other repetitive DNA loci, transposable elements are often silenced by heterochromatin formation to limit the potentially deleterious effects of such elements (Slotkin and Martienssen, 2007). Transposable elements are silenced through a wide range of chromatin modifications, including DNA methylation, histone modifications (e.g., H3K9me and H4K20me) and chromatin condensation (Slotkin and Martienssen, 2007). Similar to PAPAS-dependent recruitment of Suv4-20h2 to the rDNA, in quiescent human cells, it was reported that ncRNAs from the transposable elements *IAP* and *LINE-1* recruit Suv4-20h2 to mediate H4K20me3 enrichment and condense chromatin at transposable elements (Bierhoff et al., 2014). Such elements are also silenced through the action of small ncRNAs. For example, murine piRNAs generated from retroelements are bound to the PIWI-like protein MIWI2 and translocated into the nucleus to silence retroelements through *de novo* DNA methylation (Aravin et al., 2008; De Fazio et al., 2011). Additionally, small RNAs generated from *LINE-1* and *IAP* retroelements can regulate their epigenetic state in mouse embryos (Fadloun et al., 2013). Taken together, these studies suggest that ncRNAs help establish the epigenetic states necessary to keep transposable elements in check.

In addition to regulating chromatin compaction at transposable elements, transposon-associated ncRNAs can modulate the spatial organization of the nucleolus. For example, in HeLa cells, transcripts originating from intronic *Alu* elements (*aluRNAs*) become enriched in the nucleolus, where they interact with the nucleolin (NCL) protein and contribute to the maintenance of nucleolar structure and function (Figure 2C) (Caudron-Herger et al., 2015). Similar processes were observed in human keratinocytes and fibroblasts for *aluRNAs*, and for the related *B1* transcripts in mice. Interestingly, *aluRNAs* can somehow target other genomic loci to the nucleolus (Caudron-Herger et al., 2015), tentatively suggesting that these ncRNAs may impact spatial genome organization by establishing physical links within and outside of the nucleolus.

Given the high mutagenic potential of transposable element activity, it is perhaps not surprising that these elements have been linked to disease (Belancio et al., 2009). Transposons can promote disease through several processes including insertional mutations, deleterious non-allelic homologous recombination and the generation of *cis*-acting signals that modify gene expression (Belancio et al., 2009). It is estimated that ∼0.3% of human genetic diseases are caused by retroelements (Callinan and Batzer, 2006). For example, 15 human diseases are caused by Alu insertions while 18 germ-line diseases and 6 types of cancer are caused by unequal homologous recombination events between Alu repeats (Deininger and Batzer, 1999; Burns, 2017). In addition, *LINE-1* and *SVAs* are causative agents in numerous other human diseases (Belancio et al., 2009). In fact, the increased activity of transposable elements is a known contributing factor to neurodegenerative diseases such as Alzheimer’s disease, Aicardi Goutières syndrome, multiple sclerosis, and amyotrophic lateral sclerosis (Guo et al., 2018; Tam et al., 2019). Elevated expression of transposable elements is also a potential mechanism underlying the pathogenic development of various mental disorders including schizophrenia, bipolar disorder, autism spectrum disorders, and major depression (Misiak et al., 2019). In the context of these various diseases, it is thought that loss of heterochromatin structures may be a major contributor to the increased transposable element activity and its deleterious impact. Together, the literature indicates that ncRNAs from transposable elements can positively contribute to spatial genome organization and stability, but that losing control of such elements can disrupt genome function and promote disease.

### Centromeres

Centromeres are tandem repeats, which are largely assembled into heterochromatic structures and are important for kinetochore function and chromosome integrity. Centromeres are composed of centric and pericentric regions, which have different chromatin structures that are epigenetically established. CENP-A-containing centric chromatin is characterized by H3K4me3, while pericentric regions are enriched in H3K9me2, H3K9me3, H4K20, and HP1. Heterochromatin formation at centric and pericentric regions is mediated by NoRC, similar to heterochromatin formation at rDNA (Wong et al., 2007; Nemeth et al., 2010). In fact, the common positioning of centromeres near nucleoli may contribute to this dual role for NoRC at rDNA and centromeres (Wong et al., 2007; Nemeth et al., 2010).

Several classes of centromeric ncRNAs have been found to play a role in the establishment of centromeric heterochromatin and kinetochore function across a wide range of species. Importantly, centromeric heterochromatin is maintained by low levels of satellite repeat RNAs (Diaz et al., 1981; Trapitz et al., 1988; Rudert et al., 1995; Li and Kirby, 2003; Martens et al., 2005; Wong et al., 2007). In fission yeast, short-interfering RNAs produced by pericentromeric ‘otr’ ncRNAs help establish and maintain pericentric heterochromatin (Volpe et al., 2002), while in budding yeast, the expression of centromere-derived lncRNAs (cenRNAs) must be fine-tuned in order to maintain centromere function (Ling and Yuen, 2019). Increased cenRNA levels result in chromosome instability, aneuploidy and down-regulation of centromeric proteins while decreased cenRNA levels also result in chromosome instability. There is overwhelming evidence that centromeric- or pericentromeric-derived ncRNAs are important for the recruitment of centromeric proteins (Figure 2D) (Maison et al., 2002; Wong et al., 2007; Ferri et al., 2009; Chan et al., 2012). In *Drosophila*, centromeric SAT III ncRNAs act as a structural component of the kinetochore and are required for the recruitment of centromeric proteins (Rosic et al., 2014). In mice, lncRNAs produced from major pericentromeric satellite repeats recruit the SUMOylated form of HP1 through direct interaction with DNA at the site of their transcription (Maison et al., 2011). Murine major satellite-derived ncRNAs have also been shown to form RNA–DNA hybrids that promote the association of histone lysine methyltransferases Suv39h1 and Suv39h2 with polynucleosomes (Velazquez Camacho et al., 2017), suggesting a function for these ncRNAs in establishing heterochromatic structures. In human cells, single-stranded α-satellite RNAs are required for nucleolar localization of CENP-C and INCENP in interphase cells (Wong et al., 2007). Reducing or increasing centromeric transcription decreases the loading of several CENP proteins (Bergmann et al., 2011, 2012). In human cells and *X. laevis* egg extracts, loss of α-satellite ncRNAs reduces centromeric localization of the kinetochore protein Aurora-B and causes improper spindle attachment and chromosome misalignment (Ideue et al., 2014; Blower, 2016). Additionally, studies in maize, human cells and *X. laevis* suggest that centromeric ncRNAs stabilize CENP-C binding to DNA (Du et al., 2010; Grenfell et al., 2016; McNulty et al., 2017). Murine minor satellite repeat transcripts associate with CENP-A and regulate the structure and function of centromeres during stress and differentiation (Bouzinba-Segard et al., 2006; Ferri et al., 2009). Moreover, aberrant accumulation of these transcripts disrupts chromosome segregation, weakens sister chromatid cohesion, abrogates centromeric epigenetic signatures and results in the accumulation of micronuclei. Together, these studies reveal that the maintenance of an optimal level of centromeric ncRNAs may be important for centromeric function.

While mammalian centromeres can often co-localize with nucleoli in S phase cells, budding yeast centromeres cluster with each other at the spindle pole body, which is opposite the nucleolus (Mekhail and Moazed, 2010). Importantly, this co-localization may contribute to the cells’ ability to survive DNA double strand breaks (DSBs). Specifically, it was proposed that centromeres are released from the spindle pole body upon DNA damage induction to allow for increased chromosome flexibility and facilitate donor-acceptor locus contacts necessary for homology-directed repair (Strecker et al., 2016). The release of centromeres also drove the formation of intranuclear microtubule filaments onto which damaged DNA was mobilized by motor proteins to repair-conducive nuclear neighborhoods (Chung et al., 2015; Oshidari et al., 2018, 2019). It will be important to test whether endogenous transcription of centromeric ncRNAs contributes to this increased genome flexibility and formation of intranuclear filaments mediating DNA repair. Consistent with this possibility, the forced expression of an inducible gene integrated within a single centromere was sufficient to trigger the formation of the intranuclear microtubule filaments that are typically only observed upon DNA damage induction (Oshidari et al., 2018).

Changes to the epigenetic state of centromeres has been linked to disease (Black and Giunta, 2018). Tandemly arranged satellite repeats are prone to recombination events that can lead to chromosome rearrangements, genetic abnormalities and karyotypic abnormalities that are hallmarks of cancer (Kim et al., 2013). In addition, a study examining epigenetic signatures in ICF patients reported that, in all of the patients studied, juxtacentromeric satellite II repeats exhibited hypomethylation, tentatively suggesting that this altered epigenetic feature may underlie the chromosome fragility observed in ICF patients (Miniou et al., 1994). Centromeric repeat-associated ncRNAs have been implicated in chromatin-related changes in age and age-related diseases. There is a correlation between centromeric instability and senescence, which is potentially explained by an age-related loss of CENP-A at centromeres (Lee et al., 2010; Maehara et al., 2010; Hedouin et al., 2017). Senescence-related loss of CENP-A may be mediated by alterations to the levels of centromeric repeat transcripts, due to the fact that constitutive pericentromeric heterochromatin is decondensed in senescent cells (Swanson et al., 2013; Giunta and Funabiki, 2017). It has been directly shown that high rates of centromeric transcription can cause CENP-A translocation and mitotic arrest (Hedouin et al., 2017). Interestingly, some forms of cancer are characterized by elevated levels of α-satellite and pericentromeric satellite ncRNAs (Ting et al., 2011). These ncRNAs can form deleterious R-loop structures, which have been suggested to contribute to pericentromeric instability in several cancers (Bersani et al., 2015).

Taken together, the literature reveals numerous intersections between various types of ncRNAs and spatial genome organization in the modulation of repetitive DNA loci and their broader impact on the genome and health.

## Concluding Remarks

In this review we have highlighted roles of ncRNAs and intergenic transcription from single copy and repetitive DNA loci in the regulation of spatial genome organization. Several ncRNAs participate in spatial genome organization through several common mechanisms of action, such as chromatin looping and heterochromatin formation, while others operate through distinct pathways such as perinuclear tethering. Deregulation of spatial genome organization is associated with developmental and age-related diseases including cancer. Although aberrant expression of ncRNAs has been implicated in disease, more direct or causal links between such ncRNAs, spatial genome organization and pathobiology should be established (Palazzo and Lee, 2015). Future studies should aim to identify the exact molecular switches that induce ncRNA-dependent changes to spatial genome organization, and whether these regulatory mechanisms are conserved across evolution. Furthermore, we expect future studies to identify novel processes through which ncRNAs can regulate the relationship between genome structure and function.

## Author Contributions

NK and LO wrote the manuscript and prepared the figures. The manuscript was edited and updated by KM.

## Conflict of Interest

The authors declare that the research was conducted in the absence of any commercial or financial relationships that could be construed as a potential conflict of interest.
